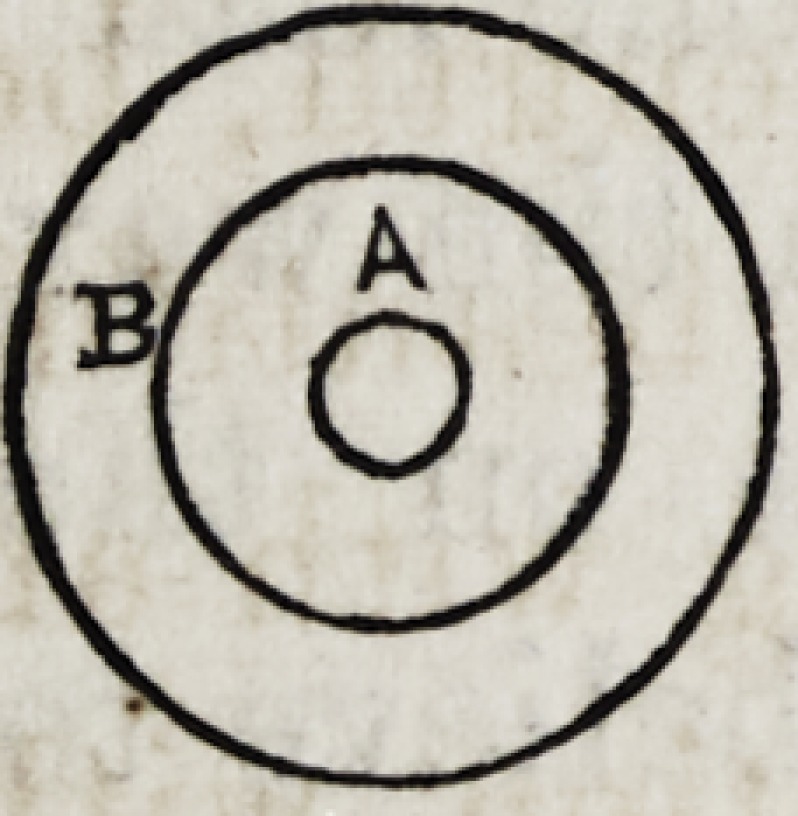# Oxygen

**Published:** 1858-07

**Authors:** R. N. Wright


### ARTICLE V,

### *Oxygen.* By Prof. R. N. Wright, M. D.

For the benefit of such readers of the Journal as may not have had it in their power to devote attention to those branches of chemistry, a knowledge of which would materially aid the intelligent dentist, we propose to publish a short series, the first of which furnishes the heading of this article.

*History.*—This substance, to the discovery of which may be referred the beginning of accurate chemical hypothesis, was observed by Dr. Priestly, of England, to be an elementary substance, about the year 1774, and a year subsequently by the celebrated Scheele, of Sweden, without any knowledge of the first discovery.

Priestly called it *dephlogisticated* air, Scheele *empyreal* air, and Condorcet *vital air*. Lavoisier, however, who conceived it to be the sole cause or principle of acidity, proposed the name it now bears, compounded of the Greek words, οξύς, *acid*, and γεννάω, *to generate.*

Oxygen gas is one of the most extensively diffused of all the elements in nature, and is a permanent gas, when not united with other substances. It forms a large portion of the water of the globe, enters into the composition of most of the soils and rocks composing the earth's crust, is a constituent of nearly all animal and vegetable substances, and exists in the atmosphere, combined with nitrogen, in the proportion of one-fifth of the former to four-fifths of the latter.

Preparation.—1st, from red oxyd of mercury.

When Dr. Priestly exposed this substance to the action of heat, he found that it was decomposed, being converted into *liquid mercury* and *oxygen gas.*

The following figure illustrates the mode of generating the gas:

A represents a glass retort, into which the oxyd is to be placed—it is supported by a stand, D; over the flame of the Argand spirit lamp, B; fastened in the clamp of an ordinary retort stand, C; the extremity of the retort is inserted in one of the openings of a double-necked receiver, E; while from the other opening a tube (F) passes to the pneumatic trough, H, in which stands a bell-glass, G. When the mechanical arrangement is complete, the oxyd to be decomposed is put into the retort, and heat is applied by means of the lamp, very soon bubbles of expanded air make their appearance and are to be allowed to escape, until the extremity of a wooden match, "red without flame, applied to the orifice, is rekindled, and made to burn with brilliancy;"* the gas may now be collected for experiment.

Using a definite quantity of the oxyd, we are enabled to ascertain its exact composition; thus, let us take 200 grains; after we have continued the heat until the retort is entirely empty, we shall find in the receiver, 185 grains of mercury, and in the bell-glass, 44 cubic inches of gas, which weighs 15 grains. We have now made an accurate analysis of this oxyd of mercury, the result proving it to be constituted thus:

| | | |
|---|---|---|
| Mercury, | . | 185 grains. |
| Oxygen, | . | 15 grains, (44 cubic inches.) |
| | | 200 grains oxyd. |

2d. The above method, although yielding a very pure gas, is not very generally resorted to—a more commonly adopted custom is the partial decomposition of *bin-oxyd of manganese,* especially where oxygen is wanted in large quantity, and *absolute* purity is not a desideratum. This substance is commonly known and sold as *black oxyd* of *manganese,* and is the highest of the three known oxyds; as may be seen by reference to the annexed list:

| | |
|---|---|
| Protoxyd of manganese, . . | Mn + O. |
| Sesqui oxyd " . . . . | 2 Mn + 3 O. |
| Bin-oxyd (native black oxyd) manganese, | Mn + 2 O. |

Strongly heated, the bin-oxyd parts with one-third of its oxygen, the remainder being a compound of the two lower

* Graham.

states of oxydation ; a change which is thus expressed by Graham : "three equivalents of bin-oxyd (131.01 parts) lose two equivalents of oxygen, (16 parts,) and leave a compound of one eq. of sesqui-oxyd and one eq. of protoxyd :"

$$3\ MnO_2 = \begin{cases} 2\ O. \\ Mn_2O_3 + MnO. \end{cases}$$

As good an arrangement as can be desired for generating oxygen from the above compound, consists simply of a refuse *gun barrel* and an empty *wrought iron mercury bottle* ; the plug being removed from the end of the tube, which is to be bent at right angles, and firmly screwed into the opening in the bottle, as represented in the annexed figure :

A represents the iron bottle, having the bent gun barrel (B) screwed securely into it, while the delivery tube (C) passes beneath the edge of the bell-glass, E, standing on the shelf of the pneumatic trough, D.

Introduce from two to six pounds of good manganese as a charge for the bottle, and, after having adapted the tube, surround it with a charcoal or anthracite fire, in a short time mingled vapors and gases will begin to come over, which will extinguish burning bodies, and must be allowed to escape ; when these vapors cease, test the gas as in the former case, and if the match, or other heated combustible, is rekindled, and made to burn brilliantly, dip the delivery tube under the water in the trough, and proceed to collect the gas.

From each pound of the best German manganese, we may expect about 1,400 cubic inches of gas, equal to about five and a half gallons by measure.

A *third process*, and one to be recommended, consists in decomposing *chlorate of potash* by heat; the annexed figure represents the form of apparatus in general use:

A is a metallic flask, (generally sheet copper,) having connection with the receiver, E, in the pneumatic trough, F, by means of the delivery tube, D; B is an Argand spirit lamp, and C, a common retort stand, supporting both the lamp and the flask.

Into the flask put about half an ounce of chlorate of potash, and expose to the heat of the lamp; the salt is decomposed with effervescence a little below red heat, yielding freely all the oxygen it contains, (in per centage, more than one-third of its weight,) leaving behind the solid, white compound, chloride of potassium. Care must be taken during the process, that the heat is not allowed to become too great, as that may result in bursting the flask.

Mr. Mitscherlich discovered, that the admixture of certain dry powders with the chlorate of potash, occasioned its decomposition at a much lower temperature than was required without them; he accordingly proposed for this purpose, the use of certain metallic peroxyds, as the oxyds of copper and manganese. Binoxyd of manganese having been previously dried at a red heat, answers perfectly well; and a mixture consisting of one part binoxyd of manganese and two parts chlorate of potash, should be kept in every laboratory, in a glass stoppered bottle, ready for use.

The following is the result of the decomposition :*

Results, { Chloride of Potassium, 1.

Oxygen, 6. eq.

or,

$$KO + C\,10_5 = \left\{ \begin{array}{l} 6\ O. \\ KC\ 1. \end{array} \right.$$

We may expect to obtain five hundred and forty cubic inches of gas from each ounce of good chlorate of potassa.

A fourth process for obtaining oxygen gas, and originally proposed by Mr. Balmain, is thus described by Graham; it consists in "heating in a retort, three parts of *bichromate of potash* in powder, with four parts of *undiluted sulphuric acid;* the gas comes off in a continuous stream, and a mixture of sulphate of potash and sulphate of sesquioxyd of chromum remains behind in the retort. The decomposition which takes place is explained in the following formula :"

*The bioxyd of manganese not being itself decomposed, but simply facilitating the decomposition of the chlorate of potash, is not taken into the account.

$KO, Cr_2 O_6$ with $4 SO_3$, give $KO, SO_3$, with $Cr_2 O_3$ $3 SO_3$ and $3O$.

"The bichromate of potash loses one-half of the oxygen contained in the chromic acid, or about 16 per cent. of its weight; one ounce of the salt yielding about 200 cubic inches of gas."

The above process, the description of which is taken verbatim from Graham, I have found extremely useful where furnaces and iron retorts could not be readily used; as a method of preparing oxygen for a table experiment, it is, I think, unquestionably the best; possessing, as it has the advantages of yielding gas with great facility, very copiously, almost without risk of accident, and sufficiently pure for most purposes.

### *Properties.*

Oxygen gas when pure, is colorless, tasteless and inodorous, with the mercury in the barometer standing at 29. 08; thermometer, 60° F., 100 cubic inches weigh 34.24; one cubic inch, therefore, weighing about one-third of a grain. Atmospheric air being taken as the standard, the specific gravity of oxygen is found to exceed it in the proportions 1105.06 to 1000. All attempts to liquify oxygen, either by cold or pressure, have heretofore entirely failed, though many experiments have been made.

Oxygen forms, with other bodies, numerous and very important compounds, all of which have the general name of oxyds. They are most commonly considered under three classes:

*The first class* embraces all such as have a general resemblance in chemical relations to potassa, soda, &c., &c., and are generally called *salifiable bases,* because capable of forming, with some other substances, a set of compounds called *salts.*

*The second class* are called *acids,* and have a strong tendency to combine with the members of the first class to form salts; as examples of this class, we might cite *sulphuric*, *nitric* and *hydrochloric acids*.

*The third class* comprises a set of bodies distinct entirely from either of the others, and seeming to possess little or no disposition to enter into combination.

In many instances we find that a body is capable of combining with oxygen in several proportions, forming a regular series, rendering necessary the adoption of some nomenclature, by which they may be distinguished from each other; this is accomplished by means of Greek or Latin prefixes, thus we say, *protoxyd*, *deutoxyd*, *tritoxyd*, *&c.* We generally find in such a series, one which possesses more strongly marked *Basiĕ* characteristics than others, and this is called the *protoxyd*. The highest known state of oxydation in a series is frequently called *peroxyd*, while an oxyd falling below the *protoxyd*, is called *suboxyd*. When there are two oxyds of the same constituents, one containing exactly twice as much oxygen as the other, the term *binoxyd* is not unfrequently used, and in case of the higher neutral oxyds, which lose oxygen more or less readily, becoming converted into *protoxyd*, the terms hyperoxyd or superoxyd are frequently employed.

Two of the most wonderful and important properties of oxygen are its effects as witnessed in *combustion* and *respiration*. All cases of combination between oxygen and other substances, are attended with the evolution of more or less heat, and whenever the temperature of any body can be raised sufficiently to make it combine with oxygen with the evolution of light and heat, the *process* is called *combustion*, and the body is termed combustible; all oxydation therefore, may be said to be combustion in varying degrees of activity, from a very low temperature to the intensely hot flame of the oxyhydrogen blowpipe.

"The economical applications of the light and heat evolved in these combinations are of the highest consequence and value, and oxydation alone, of all chemical actions, is practiced, not for the value of the products it affords, and indeed without reference to them, but for the sake of the *incidental phenomena* attending it. Of the chemical combinations, too, which we habitually witness, those of oxygen are infinitely the most frequent, which arises from its constant presence and interference as a constituent of the atmosphere. Hence, when a body combines with oxygen, it is said to be *burned;* and instead of *undergoing oxydation*, it is said to *suffer combustion;* and a body which can combine with *oxygen* and *emit heat*, is termed a *combustible.* Oxygen, in which the body burns, is then said to *support combustion*, and called a *supporter* of combustion.''*

It may be seen from the following table of Depretz, that the amount of heat produced by various combustibles during combustion, can be ascertained with absolute certainty, and an accurate estimate of their value made.

*Heat from Combustion*

| | | | | | |
|---|---|---|---|---|---|
| 1 lb. | Pure charcoal, heats from | 32° | to 212°, | 78 | lbs. water. |
| " | Charcoal from wood, | " | " | 75 | " |
| " | Baked wood, | " | " | 36 | " |
| " | Wood having 20 per ct. water, | | " | 27 | " |
| " | Bituminious coal, heats from | | " | 60 | " |
| " | Turf, | " | " | 25@30 | " |
| " | Alcohol, | " | " | 67.5 | " |
| " | Olive oil, wax, &c., | " | " | 90@95 | " |
| " | Ether, | " | " | 80 | " |
| " | Hydrogen, | " | " | 236.4 | " |

It has been pretty accurately determined, that in most instances, the amount of heat produced is proportional to the amount of oxygen consumed; which result has been deduced by Depretz, from a series of experiments, in which he burned various combustibles in a given quantity of oxygen; he found that the quantity of water heated did not in any case vary more than three quarters of a pound to a pound.

*Graham, page 299.

Besides the rapid combustion just referred to, there is another form in which the result is the same, but the progress of its development slow ; I refer to what is commonly called *slow combustion;* this is exemplified in the rusting of metals, &c. Pieces of lead, copper or iron, exposed to the action of the atmosphere, (always containing, as it does, more or less watery vapors,) will have their surfaces tarnished first, then oxydized by combination of the metal with the oxygen, which process will continue (if exposure be continued) until the entire mass of metal has become converted into oxyd throughout. The heat in these instances is generated slowly, and being immediately dissipated, there is no consequent accumulation.

Oxygen plays as important a part in respiration, as it does in combustion ; in its combination with hydrogen and carbon in the animal body being the main source of animal heat. If a small quantity of dark colored venous blood be drawn from the arm, and agitated in a vessel containing oxygen, the bright red color of arterial blood is speedily restored, which result is brought about by combination occurring between oxygen and carbon; when this chemical action happens in the living body, it is always attended with the evolution of considerable heat. Some assert and endeavor to prove that this change goes on in the lungs only, whence heat is constantly distributed to the entire system ; others, that it occurs simultaneously in the whole capillary system; that the oxygen, which the arterial blood has acquired in the lungs, is conveyed into the capillaries, where it combines with the *carbon* and *hydrogen* of the broken down tissues, forming carbonic acid and water, which are carried back to the lungs by the veins, in the dark colored blood, and eliminated by each expiration, the combustion (so to speak) of the hydrogen and carbon, by union with oxygen in the capillaries, being chiefly concerned in producing animal heat.

It might be remarked in this place, that the cause of the evolution of heat during combustion and respiration has never yet been satisfactorily made out; several ingenious hypotheses have been proposed, but all of them are purely speculative; we must be content, in the present condition of science, to accept the fact which we see and know, without being able to assign a reason for it. The presence of oxygen is not by any means indispensable for the production of heat, a great number of chemical combinations being attended with the evolution of considerable heat, indeed, we may say, that the phenomena makes its appearance to a greater or less extent, in *all* cases of chemical combinations.

*Distinguishing Characteristics.*—1. Oxygen gas is taken up sparingly by water which has been deprived of air by means of boiling or the air pump; 100 cubic inches of water so treated absorbing about 3½ cubic inches of gas; under ordinary circumstances, however, the amount absorbed is so trifling, as to be almost inappreciable.

2. If a lighted candle or taper be immersed in a glass stoppered receiver, containing oxygen, the flame is greatly enlarged from more rapid combustion, and the intensity of the light increased. Again, if the flame be extinguished leaving a small spark on the wick, the taper will be rekindled on re-introduction into the gas, a process which may be repeated successfully several times in the same gas.

3. If a watch spring be annealed by passing it through the flame of a spirit lamp, and then made into a spiral coil, by wrapping it around a cylindrical stick or piece of metal, it will burn with dazzling brilliancy on being introduced, with the loose extremity red hot, into a jar of oxygen. The best method is, after having made the coil, to tie upon the free end some sewing cotton, which is subsequently dipped into melted sulphur, a small globule of which adheres; the other end is to be secured in a bottle cork large enough to fit the neck of the receiver; fill a receiver from the gasholder, loosen the stopper, ignite the sulphur and introduce the coil; combustion of the most intensely brilliant character occurs, as the iron combines with the oxygen, forming an oxyd of that metal, which falling in hot globules, will frequently bury itself deeply in the enamel of a dinner plate, (if that be used to support the bell glass,) while others, striking the sides of the glass, will make a clean, circular perforation. This experiment, successfully performed, is one of the most strikingly brilliant within the range of chemistry.

4. A fragment of charcoal, (that made from bark being generally considered best,) suspended from a piece of copper wire, ignited and immersed in the gas, burns with great beauty, throwing off sparks in every direction; the result of the combustion in this case (if the piece of charcoal be sufficiently large to require all the oxygen) is carbonic acid gas, and which will extinguish a burning combustible instantaneously.

5. Insert in a cork, a piece of sheet copper, so bent at the lower end, as to form a sort of dependent spoon; into this put a few fragments of sulphur, ignite and introduce it into a bell glass of oxygen. Union between the burning sulphur and the oxygen takes place in this case, attended with a beautiful pale blue flame, the result of the combustion being sulphurous acid gas. That the residue in this instance really does possess acid properties, may be satisfactorily shown by pouring into the vessel containing it, one of the vegetable blue infusions, (litinus e. g.,) which will be reddened by slight agitation.

If a piece of phosphorus, about half an inch in length, be ignited and introduced into a jar of oxygen, a most beautiful deflagration is the result, the phosphorus burning with an intensely brilliant white flame, and the combination with oxygen producing large clouds of phosphoric acid, which (if the gas and bell glass are dry) is deposited on the interior in a solid form, very white, and closely resembling, in appearance, newly fallen snow flakes.

We might go on and multiply such instances as the above ad infinitum, but we have cited enough for the purpose of illustration, suffice it to say, that all substances which may be ignited, and will burn in atmospheric air, will burn with greatly increased brilliancy in oxygen gas.

Outside of the laboratory, oxygen has thus far been put to little practical use, and, in the laboratory, it is chiefly used for the purpose of increasing combustion, as in the case of the oxy-hydrogen, and other forms of blow-pipe. When a stream of the gas is passed through a spirit lamp flame, a very intense heat is produced, sufficient in most instances to fuse platinum, a still better arrangement, however, is to use a tube similar to the improved oxy-hydrogen blow-pipe nozzle, by which a stream of oxygen is made to pass through the centre of a common coal gas flame; the heat produced here is equal in intensity to the other, and the arrangement is vastly more convenient, since the combustible and the supporter of combustion, both issue from the same tube.

An inspection of the figure will render all plain:

There is a central cone of metal through which a drill is made, for the conveyance of oxygen to the centre of the flame; this central cone is surmounted by a larger one, and so adapted as to leave the space between the two cones, through which the coal gas reaches the flame, entirely unobstructed; a transverse section of the nozzle then, at the extremity, would exhibit two openings, one in the centre of the small cone, as represented by A, for the exit of oxygen, and a circular opening, B, (the open space between the two cones,) for the exit of the coal gas. By the above arrangement, we have a perfectly conical flame of carbide of hydrogen, in the very centre of which issues a jet of oxygen.

The oxygen of the atmosphere, as well as that of water, undergoes some change in properties through the agency of electricity: the former, by the passage of functional electricity through it, and the latter, when undergoing decomposition through the agency of Voltaic electricity. The change in question, has been attributed to the formation of a substance called ozone ; the existence, however, of any such substance, is purely hypothetical, inasmuch as no attempts which have been made heretofore, have successfully revealed its existence. It was supposed by Schönbein, by whom it was very carefully examined, to be a poroxyd of hydrogen, and extremely volatile.

## Figures and Tables

**Figure f1:**
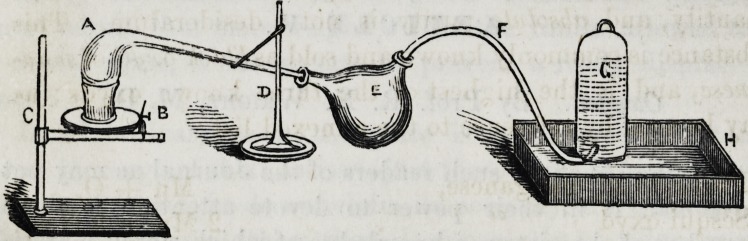


**Figure f2:**
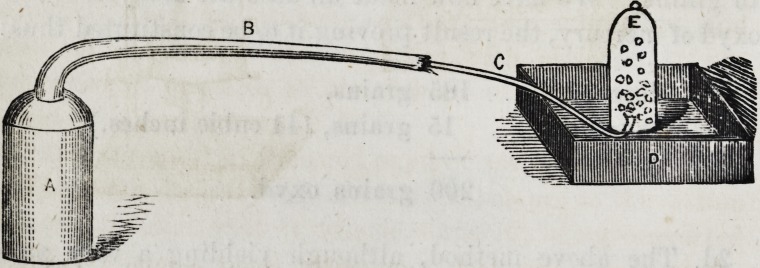


**Figure f3:**
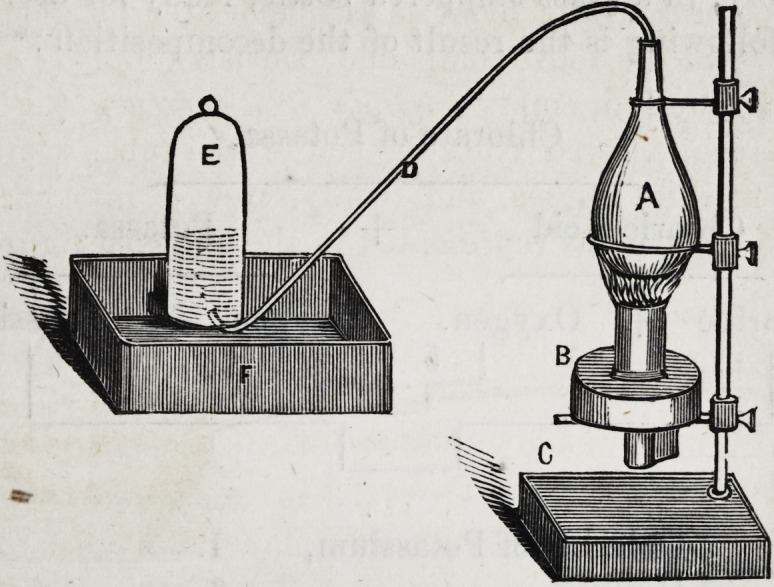


**Figure f4:**
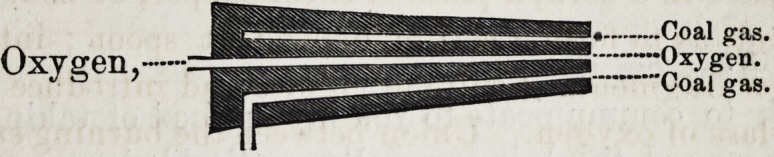


**Figure f5:**